# Safety and humoral responses to BNT162b2 mRNA vaccination of SARS-CoV-2 previously infected and naive populations

**DOI:** 10.1038/s41598-021-96129-6

**Published:** 2021-08-16

**Authors:** Shai Efrati, Merav Catalogna, Ramzia Abu Hamad, Amir Hadanny, Adina Bar-Chaim, Patricia Benveniste-Levkovitz, Osnat Levtzion-korach

**Affiliations:** 1 Research and Development Unit, Shamir Medical Center, Zerifin, Israel; 2 Sackler School of Medicine, Tel Aviv University, Tel Aviv, Israel; 3 Clinical Chemistry Laboratory, Shamir Medical Center, Zerifin, Israel; 4 Medical Management, Shamir Medical Center, Zerifin, Israel

**Keywords:** Vaccines, Viral infection

## Abstract

Since COVID-19 risk of reinfection is of great concern, the safety and efficacy of the mRNA-based vaccines in previously infected populations should be assessed. We studied 78 individuals previously infected with SARS-CoV-19, who received a single dose of BNT162b2 mRNA COVID-19 vaccine, and 1:2 ratio matched infection-naïve cohort who received two injections. The evaluation procedure included symptom monitoring, and serological tests. Among the post-infected population, the median IgG-S response after the first vaccine dose was 3.35 AU, compared to 2.38 AU after the second vaccine injection in the infection naive group. A strong correlation was demonstrated between IgG-S level before vaccination, and the corresponding responses after a single vaccine dose (r = 0.8, p < 0.001) in the post infected population. Short-term severe symptoms that required medical attention were found in 6.8% among the post-infected individuals, while none were found in the infection naïve population. Our data suggest that a single vaccine dose is sufficient to induce an intense immune response in post-infected population regardless of seropositivity. Although some short-term safety issues were observed compared to the infection naïve population, a single dose regimen can be considered safe in post-infected populations.

## Introduction

Vaccines to prevent severe acute respiratory syndrome coronavirus 2 (SARS-CoV-2) infection are considered the most promising approach for curbing the pandemic. There are several types of SARS-CoV-2 vaccines and mRNA-based vaccines were the first to be approved by the FDA with an efficacy of 94–95% for preventing a symptomatic disease^1–3^. The safety and efficacy of the mRNA-based vaccines were evaluated in prospective clinical trials on SARS-CoV-2 naïve population and the data on the vaccine usability in a previously infected population is gathered from real life case series data^4–7^.

Following a SARS-CoV-2 infection, most patients develop detectable serum antibodies to the receptor-binding domain of the viral spike protein along with associated neutralizing activities^8–15^. It was previously reported that some cases of asymptomatic and mildly symptomatic patients failed to mount neutralizing antibodies^8–11,16,17^. However, other studies indicate that the vast majority do develop detectable levels of IgM, IgG-S and IgG-N that can persist for more than six months after the acute infection^12,14,18,19^. The minimal level of antibodies required for infection immunity has yet to be determined. It is also known that in addition to protective antibodies, immunity for recurrent infections includes SARS-CoV-2-specific memory lymphocytes, including the S-antigen presenter by mRNA-based vaccine, that upon antigen reencounter are activated to generate antibodies and secrete a variety of cytokines^15,20^. It is still not clear if seronegative previously infected individuals are also at an advantage upon recurrent infection.

In addition to vaccine availability, the issue of whether to immunize previously infected SARS-CoV-2 patients is still debated since most of the vaccine related side effects have been attributed to over-activation of the immune system^3^.

The aim of this study was to evaluate the safety and efficacy of a single injection protocol of SARS-CoV-2 mRNA-based vaccine (BNT162b2) in a previously COVID-19 infected population and compare it to the standard two injection protocol given to the infection-naïve population.

## Results

### Cohort characteristics

A total of 78 confirmed post-COVID-19 infection patients, who performed a serological test after the first vaccine dose, were identified in Shamir Medical Center’s database. Of them, 45 (57.7%) had a pre-vaccination SARS-CoV-2 serology test as well. Within this subgroup, 9/45 (20%) were seronegative before vaccination.

The infection naive population consisted of 177 cases. Among this cohort, 71 (40.1%) participants had pre-vaccination serology test results, and 24 (33.8%) of them preformed a serology test after the first dose as well. Cohort baseline characteristics, demographics, and high-risk comorbidities data are provided in Table 1.

**Table 1:** Baseline characteristics.

	Post-infection	Naïve	p-value
N	78	177	
Age (y)	46 [31–60]	46 [36–59]	0.11
Age < 55	51 (65.4)	122 (68.9)	0.66
Age > 55	27 (34.6)	55 (31.1)	0.66
Male	44 (56.4)	87 (49.2)	0.34
Female	34 (43.6)	90 (50.8)	0.34
Overweight	9 (11.8)	22 (12.4)	1.00
Cancer	0 (0.0)	4 (2.3)	0.32
Diabetes	2 (2.6)	17 (9.6)	0.07
Hypertension	7 (9.2)	28 (15.8)	0.17
Heart disease	4 (5.3)	10 (5.6)	1.00
Immune deficiency	0 (0.0)	0 (0.0)	1.00
Asthma	1 (1.3)	3 (1.7)	1.00
Allergy	1 (1.3)	9 (5.1)	0.29
Chronic lung disease	1 (1.3)	0 (0.0)	0.31
Chronic liver disease	0 (0.0)	0 (0.0)	1.00
Chronic kidney disease	0 (0.0)	1 (0.6)	1.00
Hematologic disease/disorder	1 (1.3)	3 (1.7)	1.00
Chronic neurological impairment	1 (1.3)	1 (0.6)	0.52
Organ or bone marrow recipient	0 (0.0)	0 (0.0)	1.00
BMI^a^	26.1752 [24.2–29.9]	25.234 [22.5–29.1]	0.12
Time from infection (days)	116.5 [96–155]		

Data are presented as n (%); continuous data, median [IQR].

^a^The body-mass index is the weight in kilograms divided by the square of the height in meters.

### Safety and side effects

**Figure 1:**
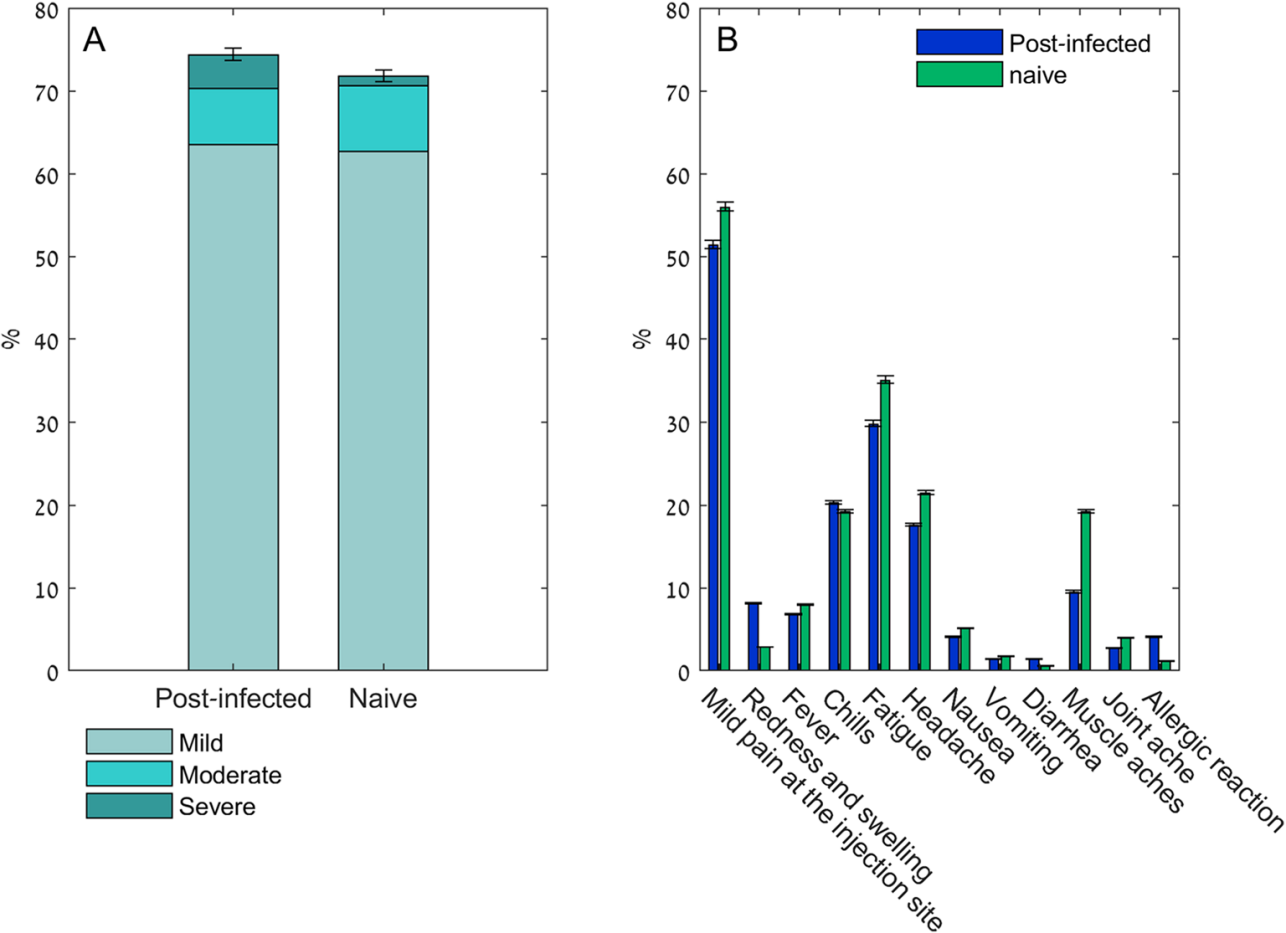
Local and systemic side effects reported after the first vaccine dose in the post-infected population (n = 78), and after the second vaccine dose in the naïve population (n = 177). (**A**) Overall side effects and symptoms in both populations (p = 0.88). (**B**) Relative frequency of each reported local and systemic side effects (a participant possibly having more than one symptom). Error bars represent 95% confidence intervals. Mild, does not interferes with daily activity; moderate, some interference with activity, or temperature > 38.5 °C; severe, prevents daily activity, temperature > 39 °C, or emergency department visit or hospitalization^3^.

We assessed the frequency of local and systemic side effects after vaccination in the post-infected and in the infection-naive cohorts. Local and systemic symptom severity was determined based on^3^. Briefly, Mild, does not interferes with daily activity; Moderate, some interference with activity, or temperature > 38.5 °C; Severe, prevents daily activity, temperature > 39 °C, or emergency department visit or hospitalization. Figure 1A shows that similar percentages of participants reported having side effects, by severity, in both cohorts. A total of 55/78 (70.5%) reported having a side effect in the post-infected cohort. Similarly 117/177 (66.1%), and 127/177 (71.8%) reported having any type of side effect in the infection-naive cohort after the first and the second injection respectively (Fig. 1A). Most common side effects were local injection-site symptoms (mild pain, redness and swelling), which occurred a few hours after the injection (Fig. 1B, Table 2). Short term allergic reactions were more pronounced in the post-infected population 4.1% compared to 1.1% in the infection-naïve cohort, as well as the overall severe symptoms that required medical attention (emergency department visit or hospitalization), 6.8% in the post-infected population, and 0.6% after the first vaccine dose, and none after the second in the infection naïve population.

**Table 2:** Vaccine-associated side effects.

	Post-infected	Naïve		p-val1	p-val2
	After first dose	After first dose	After second dose		
Mild pain at the injection site	38 (51.4)	111 (62.7)	99 (55.9)	0.040	0.340
Redness and swelling	6 (8.1)	5 (2.8)	5 (2.8)	0.097	0.097
Fever	5 (6.8)	2 (1.1)	14 (7.9)	0.029	0.799
Chills	15 (20.3)	8 (4.5)	34 (19.2)	< 0.001	1.000
Fatigue	22 (29.7)	32 (18.1)	62 (35.0)	0.095	0.314
Headache	13 (17.6)	22 (12.4)	38 (21.5)	0.430	0.402
Nausea	3 (4.1)	2 (1.1)	9 (5.1)	0.169	1.000
Vomiting	1 (1.4)	0 (0.0)	3 (1.7)	0.306	1.000
Diarrhea	1 (1.4)	0 (0.0)	1 (0.6)	0.306	0.519
Muscle aches	7 (9.5)	9 (5.1)	34 (19.2)	0.266	0.043
Joint ache	2 (2.7)	3 (1.7)	7 (4.0)	0.643	0.726
Allergic reaction	3 (4.1)	1 (0.6)	2 (1.1)	0.087	0.169
Emergency department visit or hospitalization	5 (6.8)	1 (0.6)	0 (0.0)	0.011	0.002

Data are presented as n (%); p-val1: Post-infection vs. Native dose 1; p-val2: Post-infection vs. Native dose 2.

### SARS-CoV-2 antibody responses

**Figure 2:**
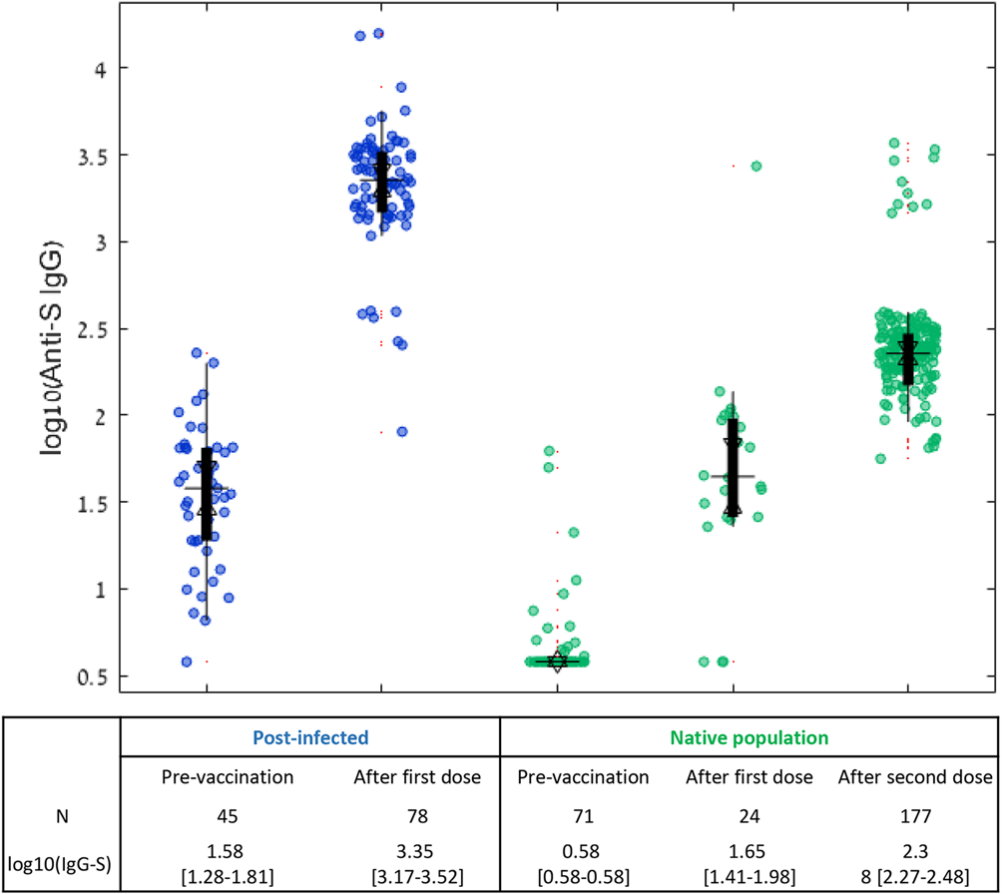
Serological response to BNT162b2 mRNA COVID-19 vaccine in post-infected individuals and naive population. The boxplot’s central mark indicates the median, and the bottom and top edges of the box indicate the 25th and 75th percentiles, respectively. p = 0.307 between the post-infected pre-vaccination group and the naïve population after the first dose, otherwise p < 0.0001.

**Figure 3:**
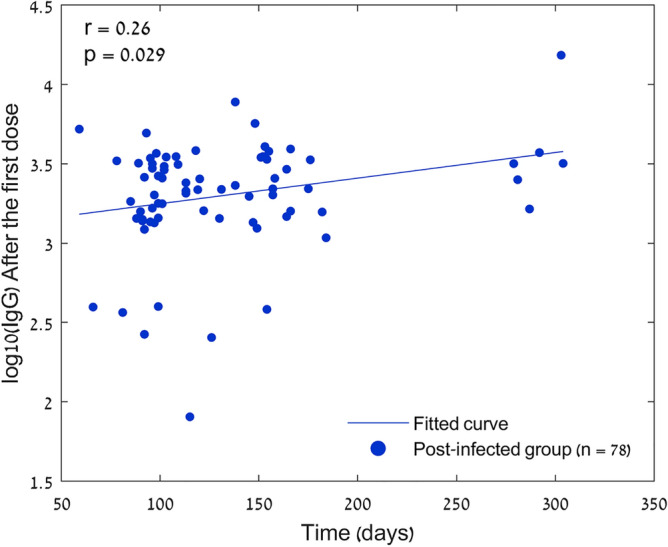
Scatterplot of the time from infection to first vaccination in days, and IgG-S responses in the post-infected cohort. r is Pearson's correlation coefficient.

We compared antibody titers of the post-infected individuals after a median of 15 days [IQR 12–18] from the first vaccine dose, with comparable 177 naive population results taken after a median of 14 days [IQR 11–17] from the second vaccine dose (p = 0.68). Among the post-infected population, the median IgG-S response after the first vaccine dose was 3.35 AU [IQR 3.17–3.52], while in the infection-naive group, the median level was 1.65 AU [IQR 1.41–1.98] (n = 24), and 2.38 AU [IQR 2.27–2.48] (n = 177) after the first and second vaccine injections respectively (Fig. 2). The time from infection to vaccination was 116.5 days [IQR 96–155]. There was no correlation between the time from infection to vaccination and antibody response levels (Fig. 3).

**Figure 4:**
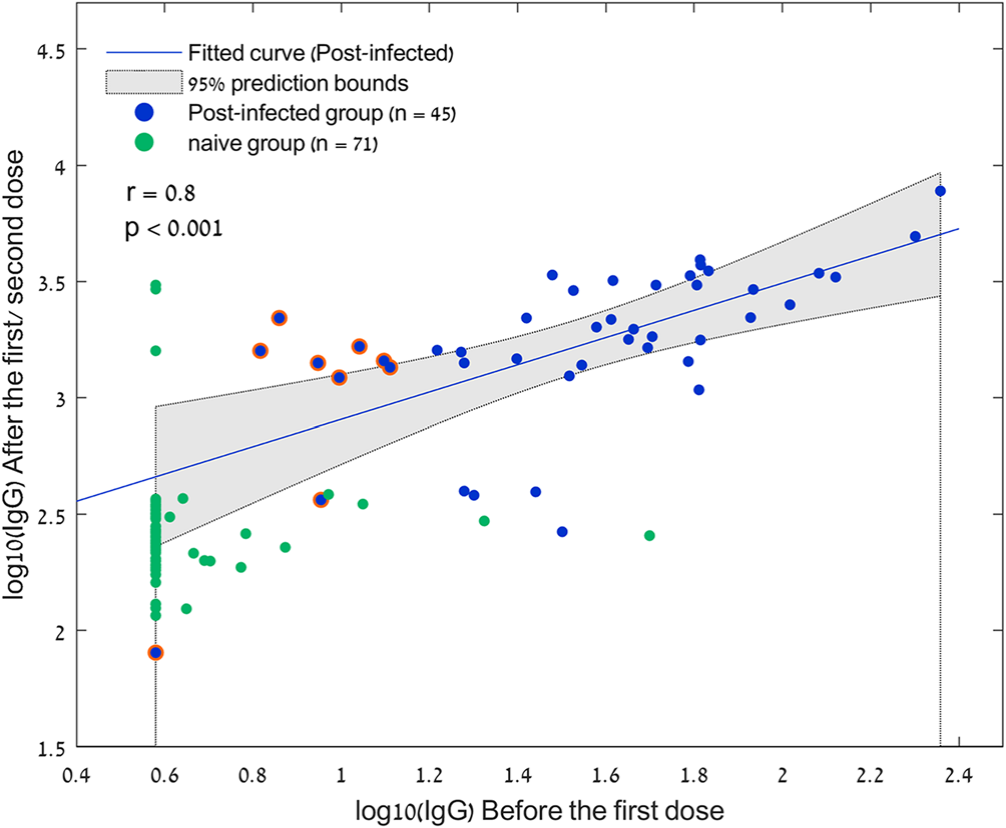
Scatterplot of the IgG-S responses before vaccination, and the corresponding responses after the first dose in the post-infected cohort (blue), and before vaccination and after the second does in the naive cohort (green). r is Pearson's correlation coefficient. The 95% prediction interval is presented in the gray area. Post-infected participants who were found seronegative prior to their vaccination are highlighted in red.

Repeated sampling analysis was performed in a subgroup of 45 post-infected participants who had also pre-vaccination antibody measurements. Among this group, the median pre-vaccination IgG-S response was 1.58 AU [IQR 1.28–1.81], and 3.25 AU [IQR 3.14–3.48] after the first dose. A strong correlation was demonstrated between IgG-S levels before vaccination, and the corresponding responses after a single vaccination dose (r = 0.8, p < 0.001) as shown in Fig. 4. Notably, the post-infected participants who were found seronegative prior to their vaccination had significantly higher titer levels after a single vaccine dose 3.15 AU [IQR 3.00–3.21] (n = 9, highlighted in Fig. 4), compared to the infection-naive group after two vaccination dosages 2.38 AU [IQR 2.27–2.48] (n = 177).

## Discussion

This study evaluates the safety and efficacy of a single mRNA-based vaccine dose in post-SARS-CoV-2 infected individuals. We demonstrate that a single dose induces a strong humoral response regardless of seropositivity in previously infected subjects. Although they had higher rates of adverse events compared to the infection-naïve population, a single vaccine dose was generally safe, and this regimen can be considered for the post-infected population.

The BNT162b2 mRNA vaccine, administered in a two-dose regimen, provides 95% protection against COVID-19 at least seven days after the second dose^1–3^. The reported efficacy after the first dose is 52%^3^. Similar to previous case series that evaluated antibody levels after a single mRNA-based vaccine dose in post-infected individuals^5–7^, our post-infected population developed, approximately tenfold higher antibody titer levels compared to the antibody levels after a second dose in the infection-naïve cohort, 2 weeks after vaccination. A linear correlation was found between the pre and post-first vaccine dose antibody titers in the post-infected population. Importantly, even among all seronegative participants who had evidence of a previous SARS-CoV-2 infection, a single vaccine dose induced higher titer levels compared to the infection naïve cohort, suggesting immune memory persistence. Specifically, due to the decay of immune memory over time, a single vaccine dose serves as a very potent booster dose for seronegative individuals who were previously infected. With the risk of reinfection increasing with time, several COVID-19 reinfection cases have already been documented^21–28^. The fact that most reinfected patients are asymptomatic or mildly symptomatic, raises the possibility that immunity is maintained and reduces the disease symptom severity even in cases of undetectable antibody levels at the time of reinfection. Here, we studied post-infected individuals vaccinated 3–6 months after the infection. Although there was no correlation between the time from the infection and antibody titer levels, a single vaccine dose induced a strong humoral response even in seronegative individuals.

The higher antibody response after a single vaccination in post infected patients is related to the acquired immune system, developed after the viral exposure. Upon the first infection, B-cells can differentiate into the plasma cells, and produce a first wave of virus neutralizing antibodies, that diminish gradually once the virus was eliminated, or develop into memory B-cells and long-live plasma cells. Memory B-cells preserve specific B-cell receptors (BCRs) of the pathogen that allows them to recognize antigen and mount a specific antibody response. When memory B-cells reencounter their specific antigen, they can proliferate and differentiate into plasma cells faster and of greater magnitude compared with the primary antibody response, and produce a more efficient secondary immune response^29^. Taken together, in post-infected populations, a single dose vaccine along with a serological test may provide an effective alternative regimen.

Local and systemic side effects were reported among BNT162b2 vaccine recipients. Reactogenicity after the first dose was characterized mostly with mild or moderate local reactions, while after the second dose, also systemic side effects were more common and severe. The frequency of severe systemic events was less than 0.9% and 2% after the first and the second vaccine injections respectively^1–3^. In this study, the reported frequency of local and moderate systemic side effect was in agreement with previous studies. However, 6.8% of the post-infection cases required emergency department visits or hospitalization due to over activation of the immune system or allergic reactions that needed medical supervision and care, while none of the infection naïve participants needed medical care. Severe vaccine related side effects were also reported in other studies of post-infected population^4,5^. This finding should be taken into consideration when vaccinating this population.

Although this study provides clear evidence regarding immune memory persistence in post infected individuals, the study has several limitations. The relatively small sample size of the post-infected population affected the infection-naïve cohort selection, due to the matching procedure, that may not represent the overall population. In addition, we studied COVID-19 post-infected individuals who were mildly symptomatic, with relatively low pre-vaccination titer levels. Finally, our study does not include information on cell mediated immunity (CMI) responses, which would provide further insight regarding the immune response, especially in post-infected seronegative individuals. Whether our safety conclusions could be generalized to previously moderate and severe COVID-19 infected patients^30^, has yet to be determined.

In conclusion, this study demonstrates that in previously SARS-CoV-2 infected populations, a single dose of an mRNA-based vaccine is sufficient to induce an intense immune response regardless of seropositivity. This vaccine-induced response correlates with the pre-vaccination IgG-S antibody concentration. The overall safety profile of the post-infected cohort is similar to the infection naive population, supporting the notion that a single dose vaccination approach can be considered in this population.

## Methods

### Study population

A retrospective analysis was conducted on a cohort of individuals, 18 years or older, who received BNT162b2 mRNA COVID-19 Vaccine (Pfizer, New York, US, and BioNTech, Mainz, Germany), and preformed serological tests, within 7–21 days after vaccination, at the Shamir Medical Center, between December 2020, and February 2021. Two population groups were identified: individuals previously infected with SARS-CoV-19, and 1:2 ratio matched infection naive population, who received two vaccination injections. Epidemiologic, demographic, pre-existing conditions data, and symptom information after each vaccine dose were collected by telephone interview. The study was approved by Shamir Medical Center institutional review board (IRB) (No. 029-21-ASF), with a waiver for informed consent. Because of the retrospective nature of the study, questionnaire completion was implied as informed consent. All research was performed in accordance with relevant guidelines and regulations.

### SARS-CoV-2 serology evaluation

COVID-19 serological tests were performed using the Liaison SARS-CoV-2 S1/S2 IgG (311450, DiaSorin, Saluggia, Italy): A chemiluminescent immunoassay (CLIA) for quantitative determination of anti-S1 and anti-S2 specific IgG antibodies using magnetic beads coated with S1 and S2 antigens. The analyzer automatically calculates SARS-CoV-2 S1/S2 IgG antibody concentrations expressed as arbitrary units (AU/ml), with a positive minimal cutoff level of 15.0 AU/ml.

### Statistical methods

We compared antibody levels and symptom responses between the two study populations of vaccine recipients. Continuous data are expressed as median and interquartile range (IQR). Independent t-tests with two-tail distribution were performed to compare variables between groups, when a normality assumption holds according to Kolmogorov–Smirnov tests. Categorical data were expressed in numbers and percentages and compared by chi-square tests. Univariate analyses were performed using Fisher’s exact test to identify significant variables. Local and systemic side effects are presented as numbers, percentages, and associated Clopper–Pearson 95% confidence intervals. Continuous parameters correlations were performed using the Pearson correlation analysis. Boxplot analysis was used to present data distribution, and to detect outliers. Matching criteria included age (in bins of 5 years), gender, and comorbidities (in count of pre-existing conditions as listed in Table 1). Hamming distance was used to compute the similarity metric. A value of p < 0.05 was considered significant. Data were statistically analyzed using the Matlab Statistics Toolbox, R2020b (Mathworks, Natick, MA).
